# Distributed Simulation as a modelling tool for the development of a simulation-based training programme for cardiovascular specialties

**DOI:** 10.1186/s41077-017-0049-y

**Published:** 2017-09-20

**Authors:** Tanika Kelay, Kah Leong Chan, Emmanuel Ako, Mohammad Yasin, Charis Costopoulos, Matthew Gold, Roger K. Kneebone, Iqbal S. Malik, Fernando Bello

**Affiliations:** 1 Imperial Centre for Engagement and Simulation Science, Imperial College London, 3rd Flr Chelsea and Westminster Hospital (Academic Surgery), 369 Fulham Road, London, SW10 9NH UK; 2 Barts and The London NHS Trust, London, UK; 3 Imperial College Healthcare NHS Trust, London, UK

**Keywords:** Distributed Simulation, Immersive simulation, Training, Collaborative design, Evaluation, Mixed-methods, Implementation

## Abstract

**Aims and background:**

*Distributed Simulation* is the concept of portable, high-fidelity immersive simulation. Here, it is used for the development of a simulation-based training programme for cardiovascular specialities. We present an evidence base for how accessible, portable and self-contained simulated environments can be effectively utilised for the modelling, development and testing of a complex training framework and assessment methodology. Iterative user feedback through mixed-methods evaluation techniques resulted in the implementation of the training programme.

**Approach:**

Four phases were involved in the development of our immersive simulation-based training programme: **(**1) initial conceptual stage for mapping structural criteria and parameters of the simulation training framework and scenario development (*n* = 16), (2) training facility design using *Distributed Simulation*, (3) test cases with clinicians (*n* = 8) and collaborative design, where evaluation and user feedback involved a mixed-methods approach featuring (a) quantitative surveys to evaluate the realism and perceived educational relevance of the simulation format and framework for training and (b) qualitative semi-structured interviews to capture detailed feedback including changes and scope for development. Refinements were made iteratively to the simulation framework based on user feedback, resulting in (4) transition towards implementation of the simulation training framework, involving consistent quantitative evaluation techniques for clinicians (*n* = 62). For comparative purposes, clinicians’ initial quantitative mean evaluation scores for realism of the simulation training framework, realism of the training facility and relevance for training (*n* = 8) are presented longitudinally, alongside feedback throughout the development stages from concept to delivery, including the implementation stage (*n* = 62).

**Findings:**

Initially, mean evaluation scores fluctuated from low to average, rising incrementally. This corresponded with the qualitative component, which augmented the quantitative findings; trainees’ user feedback was used to perform iterative refinements to the simulation design and components (collaborative design), resulting in higher mean evaluation scores leading up to the implementation phase.

**Conclusions:**

Through application of innovative Distributed Simulation techniques, collaborative design, and consistent evaluation techniques from conceptual, development, and implementation stages, fully immersive simulation techniques for cardiovascular specialities are achievable and have the potential to be implemented more broadly.

## Introduction

### Background

This paper explores the use of *Distributed Simulation* [1] as a modelling tool for the development and implementation of an immersive simulation-based training programme for cardiovascular specialities. Here, we identify gaps in the current provision of training provision for cardiovascular specialties, and in doing so outline the requirements of an immersive training programme which we addressed through the effective utilisation of Distributed Simulation.

The ICCESS (Imperial College Centre for Engagement and Simulation Science) team have developed the concept of Distributed Simulation (DS), involving realistic, accessible and portable simulated environments in recognition of various limitations involved in traditional medical simulation techniques. Drawbacks include practical issues such as the cost and expense of dedicated high-fidelity simulation centres, accessibility, and moving beyond the use of bench-top simulators that focus primarily on technical skills training [2], to more contextualised immersive training environments that can trigger a range of responses in clinicians, allowing team-based training to include human factors components, e.g. teamwork, communication, leadership and cooperation. Distributed Simulation can also allow for other innovations to be introduced such as embedding simulated patients [3, 4], thus augmenting the human interaction aspects and realism, serving to enhance the training experience further.

To date, the Distributed Simulation concept has involved several applications, from surgical training [5–8], clinician engagement workshops [9] and patient/public engagement forums [10–12]. The concept extends further through its vision to provide an approach to learning, which can be tailored to the needs of groups, and it can be used to underpin collaborative user-based design to drive forward innovations in training for emerging specialities that have to date been lacking in terms of robust development [1].

We describe how innovative concepts such as Distributed Simulation can be used as a collaborative engagement tool, involving accessible, portable and self-contained simulated environments that can be effectively utilised and applied for the modelling, development and testing of a complex training framework and assessment methodology. Iterative user feedback through mixed-methods evaluation techniques in the design process supports the concept that innovations and collaborative user-based design are key to the successful development and delivery of medical simulation training. Here, innovation and participation are inherently linked, where novel simulation techniques and tools serve to facilitate collaborative design.

### The requirements of an immersive cardiovascular training programme

In cardiovascular medicine, traditional medical simulation techniques such as bench-top/part-task simulators have been widely used for technical skills training [13–23]. Although these may be useful in the training of procedural tasks, the complexity of the clinical environment as an interactive team-based setting can be decontextualized and lost; furthermore, it has been argued that focusing on decontextualized skills only, bench-top simulators can fail to fully capture clinical performance under pressure, which is reinforced when considering that adverse events in operating rooms are often caused by failures in teamwork as opposed to technical proficiency, thus supporting the need for simulations to allow for procedure and team-based skills to be practised and assessed concurrently [24–26]. Although fully immersive simulation techniques are now considered to be one of the latest frontiers in the field [27] and commercial specialised hybrid simulated angio-suites are available, they can be prohibitively expensive and are yet to be fully utilised in the delivery of structured integrated training for procedural, technical and team-based skills. The importance of cardiovascular simulation-based learning has been recognised [28, 29], where new and existing trainees face inherent challenges in their chosen specialities related to the demands of medical service provision, reduction in training hours, and the need to keep pace with technological advancements in ever-expanding and highly competitive specialties [25, 26].

Recognising the gaps in existing cardiovascular training provision and delivery, as well as the limitations of part-task simulators, we have actively worked towards designing and implementing a robustly developed, immersive simulation-training programme for basic, intermediate and advanced cardiovascular practitioners and their multidisciplinary teams. In recognition of medical education approaches that foster the integrated acquisition of knowledge, skills and attitudes, we based our overall model on principles of complex learning and whole-task learning [30, 31]. Here, the focus is on real-life authentic tasks, team-based practices, routine tasks and non-recurrent tasks to encourage problem solving, reasoning and decision making. In specifically tailoring the model to cardiovascular specialities from the conceptual stage, it was clear that modelling the simulation framework would need to reflect challenges analogous to actual cardiovascular settings. UK-based specialist Heart Attack Centres provide emergency coronary interventions and treatments, where frontline staff of varying levels of experience face challenges in their roles: to execute complex technical procedures (routine electives or emergencies), to monitor and manage conscious patients whilst concurrently instructing MDT’s, and during any onset of crisis, to urgently identify and diagnose the complication—all against a stressful clinical backdrop with a steady inflow of emergency patients requiring life-saving treatment. A successful training programme for cardiovascular specialties would therefore require:

- A structured curriculum with clearly defined procedural and team-based learning objectives across physician experience levels in order to avoid ‘one size fits all’ approaches;
- A realistic and novel simulated training facility for individual practitioners and multidisciplinary (MDT) catheterization laboratory (cath-lab) teams, with integrated interventional simulator, and embedded patient (actor) to represent the conscious patient, actual clinical setting and team activities;
- Inclusion of a behavioural team-based framework mapped according to realistic clinical activities and engagement with patients;
- Authentic clinical scenarios for basic, intermediate and advanced level physicians that mirror real-life encounters and cases, based on the core cardiology-training curriculum [32];
- Selection and testing of training assessment tools for technical and non-technical skills;
- Accessibility for clinicians to test and provide evaluation feedback about the design of the simulated framework for collaborative design purposes.

### Distributed Simulation as a modelling tool for the development of an integrated cardiovascular training programme

Given the requirements for a holistic training programme, the novel training facility would need to cater for the needs of structured interventional procedures, combined with team-based learning objectives across trainee competency levels. The design would require iterative development, testing and refinement, and collaborative design and engagement would need to be central to the conceptual and physical development of the training programme. Multiple benefits associated with using the DS concept would allow flexibility for the programme to be iteratively designed and tested by clinicians in close proximity to their actual clinical workplace, without the need of an expensive one-off reproduction of a static angio-suite. According to Kneebone, central to the DS concept is the notion of ‘active design’, whereby key elements of the clinical environment are initially closely observed and identified by design teams and recreated through processes of ‘selective abstraction’ [33]. Here, the aim is not to faithfully recreate the entire clinical environment for simulation purposes but to recreate a functional setting that will serve to engage users—in this case as a simulation test bed to introduce, test and refine innovations in cardiovascular training techniques. In practical terms, the essential premise of DS is to effectively utilise simulation facilities that are ‘good enough’ to engage participants that are low-cost and portable and can be assembled in clinical or non-clinical locations. Accordingly, a DS angio-suite was purposely designed to capture the key components of the cath-lab (described in further detail below).

## Method

An overview of the four phases is shown in Table 1. Phases 1, 2 and 3 are presented here as they constitute the bulk of the development, mapping largely to steps 1–4 of the well-known Kern [33] framework for curriculum development, whilst phase four, corresponding primarily to steps 5 and 6, is included in the “Results” section below.

**Table 1:** Overview of the four phases

Phase	Stage	Activities
Phase 1	Concept and structure	Mapping the structural criteria and parameters using curriculum for basic, intermediate and advanced training levels
		Scenario development (*n* = 6)
		Skills assessment tools (procedural and team-based [32])
Phase 2	Modelling via distributed simulation	Training facility and simulated catherization laboratory design
		➔ ‘Test bed’ for user feedback
		Behavioural team-based framework
		➔ Clinical-behavioural-simulation trajectory
Phase 3	Testing, user feedback and collaborative design	Test cases at Hammersmith Hospital: basic to advanced level scenarios tested with cardiology trainees (*n* = 8) for feedback
		➔ Refinement of training facility
		➔ Refinement of structural criteria to include crisis management
		➔ Development of additional scenarios (total *n* = 16)
		➔ Finalisation of skills assessment tools (procedural, team-based and crisis management [37])
Phase 4	Implementation of training programme	Transition to ORcamp Training Facility, St Mary’s Hospital
		Finalisation of training day format
		Launch of the training programme
		Training sessions (*n* = 70)

### Phase 1: concept and structure: the integrated cardiology simulation framework and components

The integrated conceptual framework of the simulation training programme featured an embedded patient (actor), interventional VR simulator (*Mentice VIST* ^*®*^ ***,*** *Gothenburg* ***,*** *Sweden*), and behavioural team-based practices to enable performance and assessment of procedural and team-based skills concurrently via a portfolio of pre-tested scenarios.

#### Mapping the structural criteria and parameters for simulation-based cardiovascular training

Parameters were initially mapped out and developed using the core cardiology-training curriculum [32] as a baseline of key technical and procedural learning competencies for basic, intermediate and advanced trainees. The structural criteria was extended further to encompass management of routine elective, emergency and crisis cases, as well as stages of the clinical pathway, leading to the identification of clinical cases for inclusion. This mapping stage was vital prior to the development of scenarios, as from the outset of the process a key constraint became clear in that the pre-designed, commercially available VR simulator cases were restricted to specific technical procedures. Therefore, in order to maintain the integrated conceptual design of the programme, any development of scenarios would need to be in close conjunction with the predefined technical components of cases loaded on the *Mentice VIST* ^*®*^ simulators, e.g. clinical scenarios and complexities would need to be developed and tailored around the predefined technical *Mentice VIST* ^*®*^ simulator cases. Therefore, initially six cases (two each for basic, intermediate and advanced trainee levels), with clearly defined clinical outcomes were identified for further development into scenarios for initial testing purposes and user feedback.

#### Scenario development

Scenarios incorporated a range of human-factors competencies (e.g. teamwork, communication, leadership, decision-making and cooperation [34]). They were assembled by experienced cardiologists, tailored and structured according to the skills sets of basic, intermediate and advanced level trainees, based on realistic, authentic clinical cases, involving both routine and emergency procedures. They were also developed according to NICE, ALS and ESC guidelines in order to support existing curricula identified in the initial mapping stages.

### Phase 2: DS as a ‘test bed’ for user feedback and collaborative design

The DS angio-suite served as the ‘test bed’ to consolidate the following two phases of programme development in terms of testing, user feedback and collaborative design. This would allow refinements and finalisation of the concept and structure, prior to implementation of the training programme (phase 4).

#### Training facility design

**Fig. 1:**
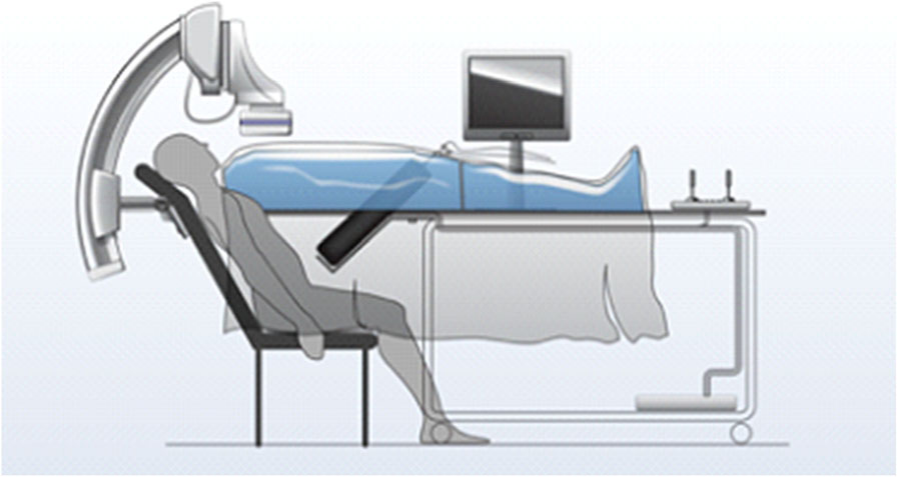
Innovative embedded patient design concept

The DS angio-suite was designed to be integrative in order to support the technical and team-based educational and training requirements. It included a robotic c-arm, a table and chair that would allow a simulated patient (actor) to be fully embedded under a mannequin during the intervention (Fig. 1). The innovative embedded patient design concept is central to the novel design of the programme, where patient-focused simulation combines trained actors within simulated environments [3]. Most cardiovascular procedures are routinely performed on conscious patients. Thus, effective communication with the patient and team is crucial before, during and after procedures and should be a vital component in skills training. Professional actors have previously been implemented in hybrid simulation and skills training, where embedding a ‘real patient’ in a training environment added to the demands of clinical performance [35, 36].

**Fig. 2:**
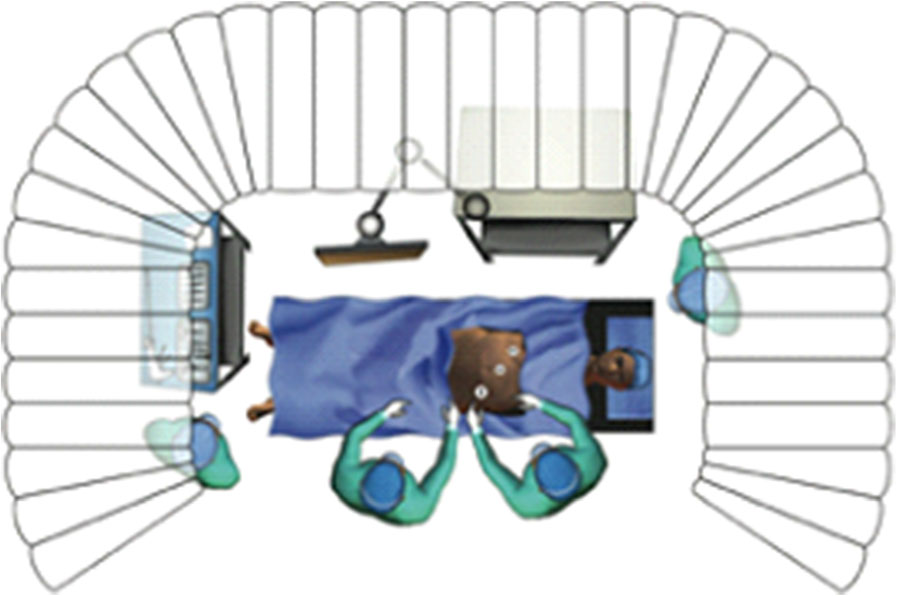
Overview of Distributed Simulation concept

**Fig. 3:**
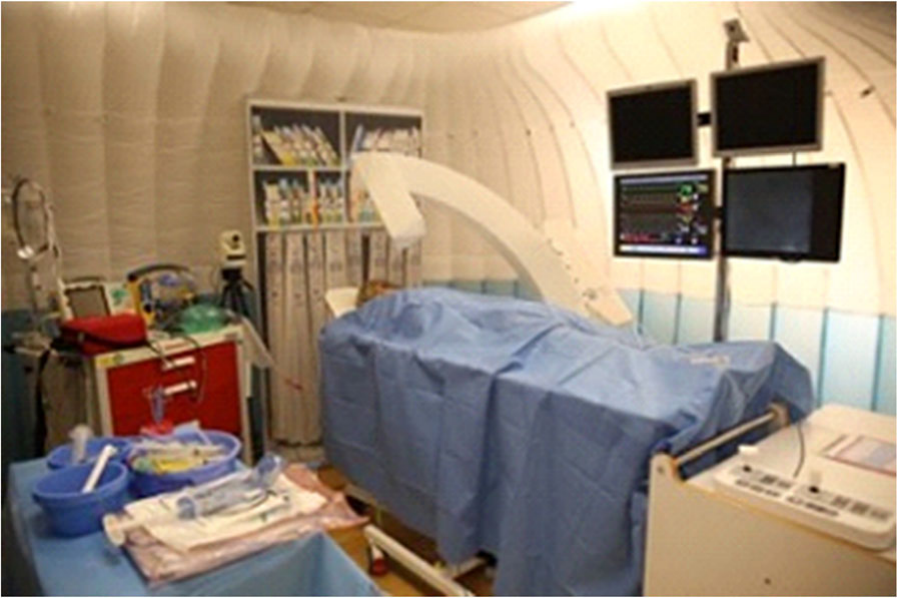
Distributed Simulation angio-suite ‘test bed’

The facility also featured an endovascular VR simulator (*Mentice VIST* ^*®*^), SimMan^*®*^ haemodynamic monitors, and key clinical apparatus and ‘props’ specific to the cath-lab, as well as space for an MDT team-interface (including nurses, physiologists, radiographers, anaesthetists and ALS team), all contained within an enclosed space with an ambience akin to that of the actual clinical environment (Figs. 2 and 3). The angio-suite also featured a separately designated control desk to avoid distracting participants from the immersive simulated setting. The interventional procedure was managed from the control desk via *Mentice VIST* ^*®*^ software, with haemodynamic traces controlled and manipulated according to the predefined scenarios using SimMan^*®*^ software.

#### Setting and location

In order to test the integrated conceptual framework of the simulation training programme, the DS angio-suite was housed in portable format at Hammersmith Hospital, Imperial College Healthcare NHS Trust with easy access to the 24-h Heart Attack Treatment Centre, thus allowing for a series of test cases to be scheduled with staff members.

### Phase 3: testing and collaborative design

#### Test cases: collaborative and iterative design via clinician involvement and feedback

The test cases commenced in November 2013. The scenarios were tested with basic, intermediate and advanced cardiology specialty trainees. A total of eight cardiology trainees (three basic, three intermediate and two advanced) were recruited across training levels from Hammersmith Hospital. Sessions typically took place in evenings after clinical hours.

#### Test case format and tasks performed

Trainees were allocated scenarios according to their experience level in advance of the sessions. On arrival, they were briefed that they would perform two to three scenarios, each lasting approximately 20–30 min, that their feedback about the test cases would inform the broader development and design of a novel, structured simulation-based training programme and that they would have the opportunity to provide detailed feedback at the end of the sessions.

Prior to commencing the scenarios, all participants received initial one-to-one instruction from an experienced interventional clinician trained in the use of the *Mentice VIST* ^*®*^, in order to familiarise themselves with the device and apparatus, including the use of guidewires and stents. All trainees also performed an initial basic test run for familiarity purposes. The ‘cath-lab team’ for the initial test cases were played by members of the research team as passive ‘plants’ to fill in the roles of nurse and radiographer, providing simulated responses when requested by cardiology operators/participants. The actors prepared in advance using the scenario material and were provided additional training about the clinical ‘symptoms’ and cues to communicate in the scenarios. They were also fitted with a discrete earpiece to receive prompts.

All scenarios started with the initial case history about the patient. Thereafter, trainees were required to follow up on the most appropriate course of action in terms of diagnosis, decision-making and type of procedure. Whilst engaged in the procedure, the simulation faculty introduced haemodynamic complexities and difficulties from the control desk, simultaneously instructing the actor to report corresponding cues via microphone and earpiece. Scenarios were not scripted but were designed to evolve according to trainees’ decisions and actions. All scenarios were video recorded.

#### Evaluation and user feedback

A mixed-methods approach was employed to evaluate user feedback, involving a quantitative survey featuring scale items (1 = strongly disagree, 5 = strongly agree) to evaluate the realism of the integrated simulation framework and components, and perceived educational relevance, including feasibility of using the simulation format and framework for training purposes.

A semi-structured interview was also conducted at the end of the sessions in order to capture more detailed feedback about the realism and utility of the simulation framework and components, including proposed changes and scope for further development for training purposes. The semi-structured interview schedule was developed according to a pre-defined thematic framework using evaluative framing questions to explore:

- Realism of the integrated simulation training framework: scenarios, technical procedure, simulated patient, multidisciplinary team and behavioural components, case flow and crisis management, the simulated angio-suite and overall experience
- Educational relevance of the training programme: overall utility across training levels, relevance to junior, intermediate and senior trainees and overall educational relevance.

## Findings

### Quantitative feedback about the integrated simulation framework and components

**Fig. 4:**
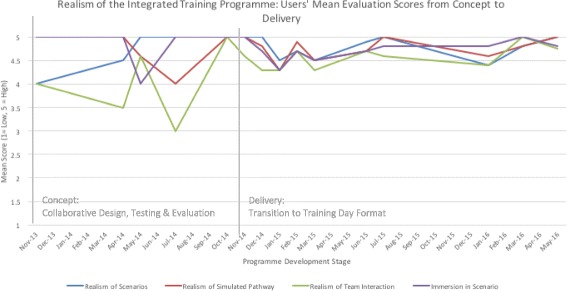
Realism of the integrated training programme: users’ mean evaluation scores from concept to delivery

**Fig. 5:**
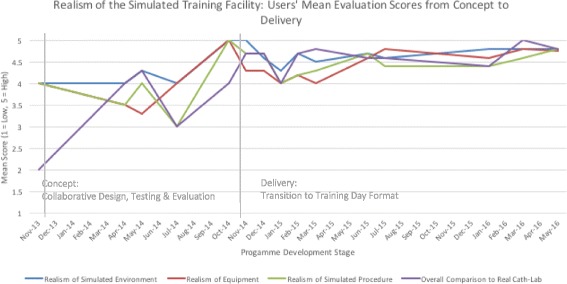
Realism of the simulated training facility: users’ mean evaluation scores from concept to delivery

**Fig. 6:**
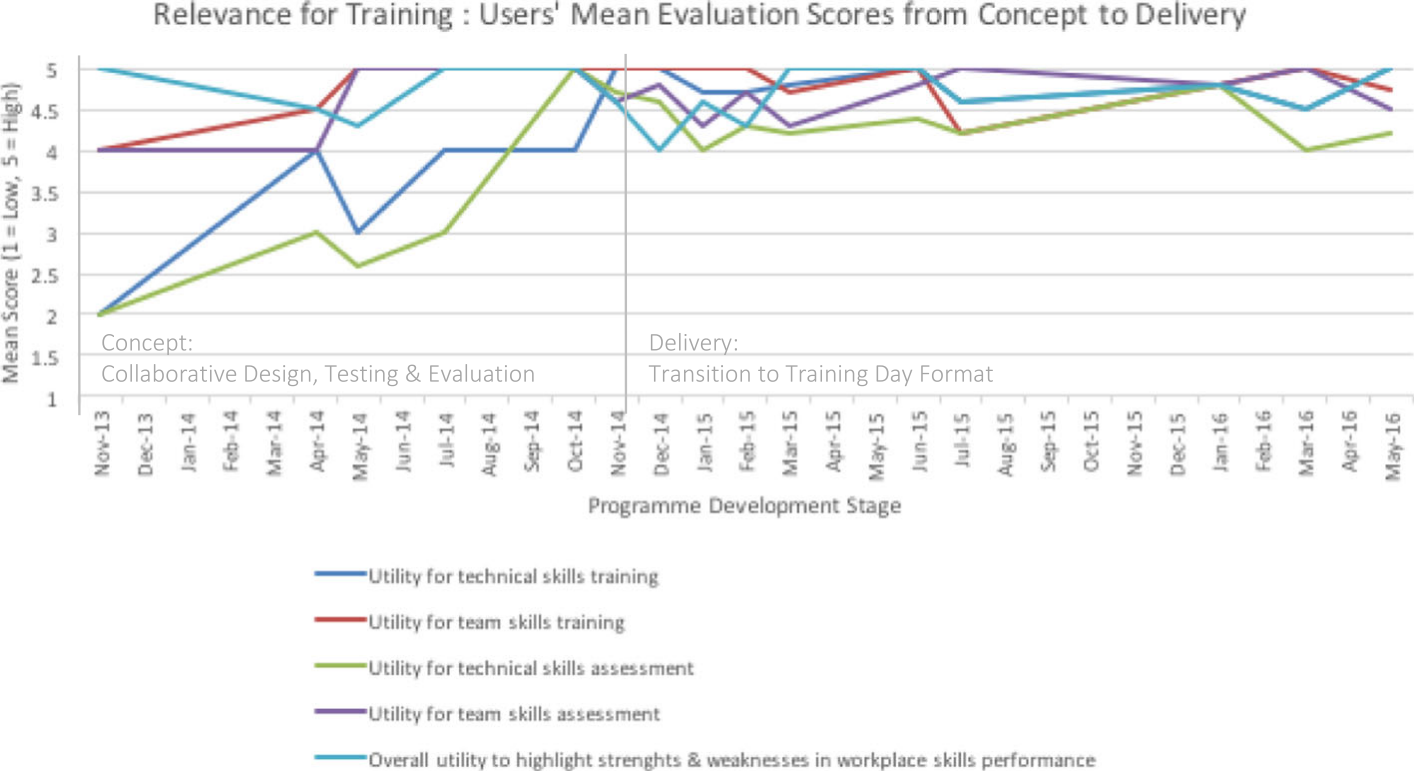
Relevance for training: users’ mean evaluation scores from concept to delivery

The users’ mean evaluation scores for realism of the simulation training framework, realism of the simulation training facility and relevance for training are presented in Figs. 4, 5 and 6. Given that refinements were made iteratively based on initial test cases (*n* = 8) and user feedback over time, mean scores are presented alongside feedback throughout the development stages from concept to delivery (Nov 2013–May 2016), including implementation stages of the training programme (*n* = 62, who had enrolled and participated in the programme) for comparative purposes.

Although mean score ratings ranged between average and high for realism of the simulation training framework, realism of the training facility and relevance for training, the lower and fluctuating mean scores were given during the initial testing and refinement phases through to the implementation phase, with incrementally higher ratings after implementation. In terms of components, the scenarios, team interaction and realism of the pathway demonstrated scope for improvements, although this did not appear to detract from overall immersion. Similarly, the realism of the training facility was scored average to high for realism of the simulated environment and equipment, with markedly lower ratings for the simulated procedure and comparisons to the actual cath-lab. In the initial testing phase, the lower ratings about the realism of the procedure were reinforced in terms of overall perceived utility of the framework for training and assessment of technical skills, while ratings for training and assessment of team skills were higher. However, participants felt that the overall format had good potential to highlight their strengths and weaknesses in workplace skills performance.

### Qualitative feedback about the integrated simulation framework and components

The qualitative user feedback data was coded according to a pre-determined, a priori thematic framework, derived from the research aims and structured interview schedule, whereby feedback was classified according to themes [37]. Alignment of the coding with the themes was independently checked by a second researcher experienced in qualitative research methods, with perfect agreement of 100%.

The qualitative feedback served to augment the quantitative evaluation, providing further clarity as to the realism and relevance of the training framework and facility. Exemplar extracts are presented by theme in Tables 2 and 3.

**Table 2:** Qualitative user feedback for realism of the integrated simulation training framework

Theme	Exemplar extracts
Scenarios	“Good flow to scenarios, starting with the procedure, then going on to the crisis” (P1, intermediate)
	“Several challenges are thrown into the scenarios which work very well in learning how to react and how quickly” (P1, intermediate”)
	“The scenarios are key to these simulations…talking through what you plan to do as you’re doing it…for training purposes it’s excellent” (P3, senior)
Technical procedure	“The feedback from the simulator didn’t feel very accurate…but the scenarios are designed around this issue which is really useful, and offers more benefits for training” (p5 junior)
	“There were some technical issues with simulator…handling the guidewire felt odd…once you get into it its ok” (P3, senior)
	“[The interventional VR simulator] feels different to the real procedure…different sensitivity…but the scenario allows adaption to this” (P4, intermediate)
Simulated patient	“Very realistic chatter, but how these interactions are handled by trainees will vary according to individual differences; I can’t always deal with talking to a patient and doing the procedure, so this is good practice” (P4, intermediate)
	“Having a real patient instead of a mannequin makes it more realistic” (P2, senior)
	“I got a lot of feedback from the patient… very realistic” (P3, senior)
Multidisciplinary team and behavioural component	“Having an actual nurse and radiographer…the actual team would make it even more realistic” (P5, junior)
	“Having to manage team members is key to these simulations…I think clinical feedback from a real cath-lab team would add to the realism and makes it feel more immersive” (P4, intermediate)
	“Having non-clinical stand-in team members is not realistic. Feedback from the team is very important…you need real cath-lab members in the team” (P3, senior)
	“Having a fully operational team would make it more realistic, i.e. radiographer, runner nurse…” (P2, senior)
Case flow and crisis management	“The crisis scenarios worked very well. The traces were very realistic” (P4, intermediate)
	“Having the crisis equipment, defibrilliator… makes it feel very realistic” (P1, intermediate)
	“The crisis was very realistic and close to real life in real PCI’s” (P2, senior)
Simulated angio-suite	“In terms of simulating an angio-suite it’s quite realistic…I think actually being involved in the scenarios and experiencing that makes it realistic” (P5, junior)
	“It’s quite realistic, relatively immersive…but there will always be a schism between reality and simulation….the patients’ torso is a bit too long so she was far too away while I was operating…I couldn’t always hear her” (P1, intermediate)
	“Having a team and task is what makes it more immersive…it means you focus less on the little things like equipment which aren’t necessarily that accurate…” (P2, senior)
Overall experience	“Simulating everything together, the pressure…the team skills, communication and crisis…to know what to do when something is going wrong, to get the right decision making…I found it more challenging and useful than the technical part which didn’t feel that accurate” (P5, junior)
	“Very realistic in terms of the whole experience: doing the procedure, talking to the patient, handling the team” (P1, intermediate)
	“Very much the same as what actually happens, and the workload in the cath-lab” (P1, intermediate)
	“I felt immersed…responding to traces, talking to the patient, interactions with team” (P4, intermediate)
	“I felt immersed in the scenario. There’s a patient there speaking to you. It felt serious and I responded to it accordingly (P2, senior)

**Table 3:** Qualitative user feedback for educational relevance of the training programme

Theme	Exemplar extracts
Overall utility across training levels	“Scenarios can be made more complex, challenging and difficult for different experience levels…” (P5, junior)
	“All trainees would want to take part in simulation based training of this nature…it’s cath-lab emergency training” (P4, intermediate)
	“With this design, scenarios can be tailored for trainees with different levels of ability and expertise” (P1, intermediate)
	“There’s a good range of scenarios for different training levels…scenarios can be made complex for more senior trainees…” (P2, senior)
Relevance to junior trainees	“This is really useful for staff starting out in the cath-lab to have training on crisis scenarios, and gaining familiarity on how to handle this” (P5, junior)
	“ST3’s should go through this as it’s good experience in instructing and managing teams and good experience in how to handle clinical scenarios and emergencies, when things go wrong…It’s important for encountering situations in the cath-lab if trainees have not done many procedures” (P1, intermediate)
	“The scenarios might be diagnostically challenging for juniors but very helpful for when first starting out. It’s not too advanced for ST3’s…in reality trainees are thrown into the deep end from the start...I would have liked to have gone through this before starting as an ST3…” (P4, intermediate)
	“This would be really important for juniors who are always with a consultant, who make decisions for them, so they may not necessarily absorb what happens” (P3, senior)
Relevance to intermediate and senior trainees	“Theoretically, intermediate and advanced trainees should have these skills already, but I think even for someone more advanced this would be good” (P5, junior)
	“This training would be good for ST5’s when they decide what they want to specialise in… for intermediate trainees the scenarios are very good as they allow them to improve their skills and do these scenarios as first operator that they wouldn’t do in real life” (P4, intermediate)
	“For senior trainees who are exposed to these situations in real life…. they should know how to handle them, but it’s good to practice acute scenarios and rare cases ie “things that could go wrong”. It doesn’t matter how senior an operator is…for rare complications that don’t happen often, this simulation is very useful too. Like perforations don’t happen very often, so this is good practice” (P2, senior)
Overall educational relevance	“I would 100% endorse these simulations for a training programme from the outset of training…it should be compulsory. Communication skills, team skills, keeping calm…. making the right decisions, it’s all there” (P5, junior)
	“Overall it was helpful, interesting and challenging, in terms of not necessary knowing what the right course of action is when things go wrong…out of the comfort zone” (P1, Intermediate)
	“Good scenarios and training kit for communicating with the team, managing difficult scenarios & situations in the cath-lab and practicing complications that don’t happen very often” (P2, senior)
	“I was in the old training system and never went through anything like this before. It’s a really good way to teach” (P3, senior)

#### Realism of the simulation training framework, components and facility

From the outset, users expressed that they had felt very immersed in the simulations owing to the scenarios themselves, which they felt were well designed in terms of case flow and clinical realism through the manipulation of haemodynamic traces. Overall, feedback for the holistic and integrated design of the simulations was very positive—trainees felt that ‘everything was happening together’ as in the actual clinical environment. However, senior trainees recognised that the interventional simulator and technical components, e.g. haptics and feedback did not feel realistic, but they felt engaged enough in the simulations as there were multiple layers of activity such as the ‘chatting’ patient and dynamic haemodynamic changes. Junior trainees, however, felt more engaged in the technical procedure and indicated that it felt realistic. Senior trainees felt that the constraints posed by the technical procedure may not necessarily detract from the simulation experience as scenarios and team-based elements had been designed around this issue. They reflected that, with the limitations of the interventional simulator in mind, the focus of the simulations should be less on procedural components and more on team-based elements, particularly crisis, emergency training and ‘what to do when something goes wrong’.

Trainees stated that the simulated patient was extremely realistic, akin to the real clinical context in which patients are typically conscious during procedures. They felt that inclusion of a ‘live’ patient was key to executing the simulations as opposed to a mannequin; they indicated that at times they felt distracted from the technical parts of the procedure by the patient’s ‘chatter’ as in real clinical cases, whilst others noted that the patient provided cues as to her/his clinical status and well-being, which is a vital issue in real case handling and decision making. However, although inclusion of the ‘live’ patient received highly positive feedback, trainees indicated that the overall set up was not realistic, as the patient’s torso was extremely long due to the original design of the table and placement of the simulator, which did not resemble a real patient. Furthermore, in supporting the team-based relevance of the simulations, trainees felt that having real cath-lab team members within the scenarios would enhance the training experience considerably as they would provide realistic clinical feedback, as opposed to having ‘stand-in’ plants unversed in the correct clinical parlance.

#### Relevance of the integrated simulation framework for training purposes

Users felt that the simulation framework was excellent for training purposes, and particularly relevant to crisis and emergency training through the introduction of difficulties and challenges. They felt that the design itself was overall highly innovative and could be tailored and applied to the skills sets and experience levels of trainees. In doing so, trainees provided recommendations for additional scenario components and clinical cases worthy of inclusion in the training programme. Therefore, overall, junior, intermediate and senior trainees recognised the utility and relevance of the training framework for themselves and their peers, with senior trainees reflecting that they would have certainly benefitted from such a training experience earlier on in their training.

### Refining the integrated simulation framework and training facility

The user-based evaluation informed the next stages of programme development, to refine key components of the simulation framework and physical set up, as well as the development of 10 additional scenarios for inclusion in the implementation phase of the programme (*n* = 62).

#### Refinements to team-based elements

Based on user feedback, cath-lab nurses and radiographers were recruited for the simulation test cases to enhance realism in terms of clinical dialogue and feedback during scenarios. The team-based interface was also extended to incorporate ALS resuscitation teams and anaesthetists at critical stages of the crisis scenarios [38].

#### Refinements to the embedded patient, simulator and mannequin torso set up

User feedback from the early test cases indicated that the patient set up did not reflect an actual human shape, with some distance between the patient’s head and the operating position. This issue was due primarily to the overall design and length of the *Mentice VIST* ^*®*^ simulator, the placement of which meant that the sheath entry point was some distance away from the patient. Therefore, the more slimline and compact *Mentice VIST-C* ^*®*^ simulator was procured and integrated with the DS angio-suite. In order to develop the crisis-components of scenarios as recommended by the user feedback, a mannequin torso was also integrated within the DS angio-suite to allow CPR during training. The DS mannequin legs were replaced to provide more realistic palpation for trainees). When fully draped, these refinements enhanced the realism of the human-like body with embedded patient and integrated simulator, with improvements to the sheath entry point and operating position. As the test cases progressed, further innovations were introduced including realistic skin and puncture site in order to allow trainees to check the puncture site during complications in complex cases such as retroperitoneal bleeding.

The physical simulation model was refined iteratively as further innovations were introduced. For example, the DS approach allowed anaesthetic team-based components to be tested, and subsequently the upper body of the SimMan 3G^*®*^ was incorporated into the physical set up, adding realistic breathing, as well as an advanced airway head for anaesthetic training purposes if required.

### Phase 4: transition to training day format and implementation of simulation-training framework

After the test cases, due to geographical convenience and space availability, the implementation of the training programme involved a transition period from Hammersmith Hospital to the Surgical Innovation Centre, St Mary’s Hospital, involving use of the ORcamp (Orzone AB, Gothenburg, Sweden) facility. This relocation allowed space provision for the training day format, including a separate debrief room where trainees and consultant assessors could view simulations and provide subsequent feedback and assessment. The move required each portable component of the pre-tested DS model to be transferred to the ORcamp simulation training facility (Table 4). In addition, the DS control desk was upscaled into a ‘control hub’ in order to successfully cater for the expanding requirements of the training day format. It is important to emphasise that the simulation setup and scenarios are not restricted to any particular location, having been subsequently used in other settings such as conference venues.

**Table 4:**
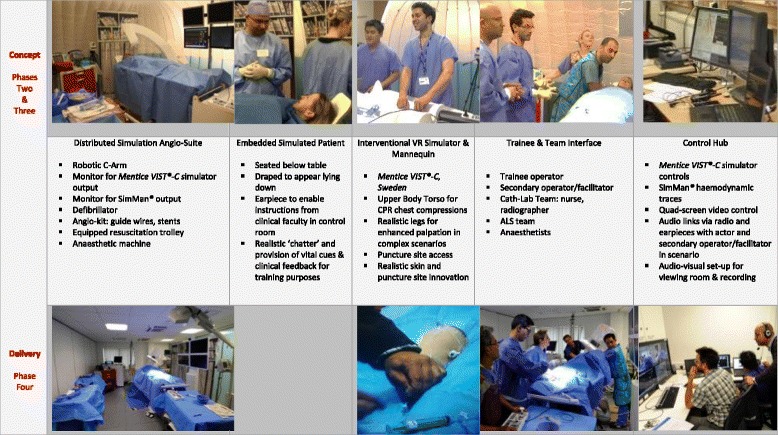
Stages of development—from concept to delivery

The Imperial Cardiovascular Simulation Training Programme (iCAST) was launched in November 2014. Twelve training days have been successfully delivered between November 2014 and May 2016, with 70 trainees to date (8 reported test cases; 62 trainees have enrolled and have participated in the training programme).

#### Training day format

Each training day involves 6–8 trainees. Scenarios are pre-allocated to trainees according to their skills level. Trainees undergo simulation scenarios followed by a structured debrief lead by consultant assessors, where trainees reflect on their technical performance and their interactions with the cath-lab team and simulated patient.

#### Integrated performance and skills assessment

Within the training format, procedural skills are assessed in conjunction with team-based skills (communication, leadership, team-working and decision-making) and crisis management skills. All assessment tools used have been validated in previous studies [13, 34, 39].

## Discussion

In this paper we have described the effective use of the innovative Distributed Simulation concept to: (a) recreate the coronary context for simulation purposes and (b) as a collaborative engagement tool through clinician-based user feedback to develop, test, model, refine and implement an integrated, fully immersive simulation-based training programme for frontline healthcare staff. The programme aims to enhance existing training by providing an integrative approach to the practice and assessment of clinical procedural skills, team-based skills and crisis management. The resulting simulation programme has received positive feedback in its pilot phase [40] and has been deemed applicable to various levels of skill development and training, as well as practitioner experience from basic to advanced levels.

The essence of Distributed Simulation lies in its functionality and application wherever and whenever it is required [1], thus addressing some of the key current challenges in medical training: cost, accessibility and positive engagement with frontline clinical staff. We have demonstrated how innovation and participation go hand-in-hand, where modelling techniques and collaborative design can be used in a cost-effective manner to robustly develop realistic and meaningful learning settings tailored for the requirements of individual practitioners and teams. Here, the DS concept was pivotal in avoiding the development of an expensive, static one-off training facility that may have proved unfeasible for the innovative training framework design, with the cost of the simulated angio-suite (£25k to £50k) being only a small fraction of an equivalent fixed facility (£250k to £500k). Instead, the DS provided clinical teams with accessibility to a test-bed angio-suite in order to test and refine the integrated simulation training framework and related components. Iterative testing and refinements to the framework over time are reflected in the overall mean evaluation scores of trainees, with an incremental rise in ratings as to the realism of the integrated framework, training facility and perceived relevance for training and educational purposes corresponding to design refinements. Here, the initial fluctuating average to high evaluation ratings in the test-cases reflects the capability of the DS test bed to introduce and test new layers of complexity, including team-based elements, procedural complexity and difficulty, and improvements in physical design based on user feedback. Key to the engagement with users for successful collaborative design and outputs is the consistent inclusion of structured mixed-methods evaluation techniques from the outset, including qualitative interviews to contextualise and augment quantitative survey findings, which may not fully capture the true meaning and depth of responses, particularly about integrative designs. For example, although the scenarios initially received low mean ratings, the interviews revealed them in a positive manner. Therefore, they remained unchanged and were subsequently successfully used.

Through close collaboration and engagement with the clinical community we have been able to understand and assimilate complex knowledge structures in order to identify learning needs. Although technological solutions are often considered to be the key drivers advancing simulation, our user-based evaluation feedback suggests that the interventional VR simulator may not always feel realistic amongst senior practitioners, yet this did not detract from the immersive scenarios or the broader context of ‘everything happening together’ as in the actual cath- lab environment. In reality, clinicians work in complex environments and need to integrate several skills sets. The novel simulation-training framework derived by this research was designed to provide this holistic approach to simulation training and skills assessment, drawing together the multiple strands of technology, team-based interactions, patient perspective and patient interaction. Thus, whilst technical simulation solutions are vital to keeping pace with rapid advancements in cardiovascular procedures, our user evaluation evidence suggests that there is a need and scope for technical simulators to be contextualised through innovative approaches such as DS. By aligning technology, simulator development and behavioural interaction with educational design, the simulation learning experience can effectively cater for complex learning needs. Our incorporation of a real person as a simulated patient alongside an interventional VR simulator is very innovative—interacting and conversing with a ‘real’ patient whilst performing a complex technical procedure can trigger a range of responses from trainees that are vital for training [3], with multiple implications for skills assessment and designing interventions for effective communication. A further learning need that the simulation framework serves is that of effective communication and teamwork, particularly in multidisciplinary team contexts, where our work has identified gaps in current training provision for anaesthetists who have reported negative experiences in the cath-lab, often due to issues related to teamwork and communication [38].

With regards to future implementation and relevance for education and training, applications of the integrated simulation-training framework are wide-ranging: to date, it has been successfully applied to the skills sets of individual cardiologist practitioners with passive teams (e.g. individual skill development decision making) and entire consultant cardiologist-led MDT teams (e.g. team skill development and decision making). In terms of cross-speciality application, we propose that the robust simulation training framework can be successfully tailored and applied to other vascular specialities through collaborative-engagement and design, e.g. endovascular, neurovascular and interventional radiology.

## Conclusions

Although technical skills training for procedures and technological solutions are often considered to be key to advancing simulation for cardiovascular training, our novel and innovative exemplar shows that for the users themselves, immersion in realistic team environments that reflect their actual clinical settings—including technical skills performance—is deemed most relevant for training. Through the application of innovative Distributed Simulation techniques, collaborative user-based design, and consistent evaluation techniques from conceptual, development, delivery and implementation stages, we have shown that fully immersive simulation techniques for cardiovascular specialities is achievable and has the potential to be implemented across vascular specialities.
